# Desmoplastic small round cell tumor: from genomics to targets, potential paths to future therapeutics

**DOI:** 10.3389/fcell.2024.1442488

**Published:** 2024-07-30

**Authors:** Justin W. Magrath, Madelyn Espinosa-Cotton, Dane A. Flinchum, Shruthi Sanjitha Sampath, Nai Kong Cheung, Sean B. Lee

**Affiliations:** ^1^ Department of Pathology and Laboratory Medicine, Tulane University School of Medicine, New Orleans, LA, United States; ^2^ Department of Pediatrics, Memorial Sloan Kettering Cancer Center, New York, NY, United States

**Keywords:** DSRCT, pediatric cancer, sarcoma, fusion oncogene, targeted therapy

## Abstract

Desmoplastic Small Round Cell Tumor (DSRCT) is a highly aggressive pediatric cancer caused by a reciprocal translocation between chromosomes 11 and 22, leading to the formation of the EWSR1::WT1 oncoprotein. DSRCT presents most commonly in the abdominal and pelvic peritoneum and remains refractory to current treatment regimens which include chemotherapy, radiotherapy, and surgery. As a rare cancer, sample and model availability have been a limiting factor to DSRCT research. However, the establishment of rare tumor banks and novel cell lines have recently propelled critical advances in the understanding of DSRCT biology and the identification of potentially promising targeted therapeutics. Here we review model and dataset availability, current understanding of the EWSR1::WT1 oncogenic mechanism, and promising preclinical therapeutics, some of which are now advancing to clinical trials. We discuss efforts to inhibit critical dependencies including NTRK3, EGFR, and CDK4/6 as well as novel immunotherapy strategies targeting surface markers highly expressed in DSRCT such as B7-H3 or neopeptides either derived from or driven by the fusion oncoprotein. Finally, we discuss the prospect of combination therapies and strategies for prioritizing clinical translation.

## 1 Background

Desmoplastic Small Round Cell Tumor (DSRCT) is an extremely rare but aggressive pediatric sarcoma most commonly originating in the peritoneal or pelvic cavities (Gerald and Haber, 2005). DSRCT is characterized by the t (11; 22) chromosomal translocation which leads to the creation of the oncogenic transcription factor EWSR1::WT1 (Ladanyi and Gerald, 1994; Gerald et al., 1995; Gerald and Haber, 2005). The DSRCT incidence rate differs by sex with an age-adjusted incidence rate of 0.22 per million in males and 0.05 per million in females within the United States (Waqar and Ali, 2022). DSRCT is highly metastatic and commonly presents with tens to hundreds of tumor nodules within the peritoneal lining at diagnosis, which poses a unique challenge for complete surgical resection (Stiles et al., 2018; Xiang et al., 2020). DSRCT derives its name from its histologic appearance which includes nests of small round cells imbedded in fibrous stroma. The cell of origin is unknown and DSRCT cells stain positive for a variety of tissue markers including those from neuronal, epithelial, mesenchymal, and myogenic lineages (Chang, 2006). While there is no standard treatment, patients commonly receive multimodal therapy including a combination of surgery, radiotherapy, and chemotherapy (Lal et al., 2005; Hayes-Jordan et al., 2016; Bulbul et al., 2017; Subbiah et al., 2018; Honoré et al., 2019). Despite this aggressive treatment, patient prognosis remains poor with a 5-year survival rate of only 15%–30% (Kushner et al., 1996; Lal et al., 2005; Subbiah et al., 2018; Honoré et al., 2019). Therefore, novel targeted therapies are urgently needed to improve patient prognosis. Here we review the recent developments in DSRCT including the establishment of new research tools, improvements in the understanding of underlying DSRCT biology, and promising therapies that have been investigated preclinically.

## 2 Model and dataset availability

As a result of DSRCT’s scarcity, sample and model availability have been a limiting factor in understanding DSRCT biology and developing effective therapeutics (Lettieri et al., 2014; Stiles et al., 2018; Gedminas et al., 2022a). Nishio et al. established the first DSRCT cell line (JN-DSRCT-1) (Nishio et al., 2002) in 2002 which remained the only commonly available DSRCT cell line for nearly 2 decades. However, recent efforts have led to the establishment of a plethora of new DSRCT cell lines (BER-DSRCT, BOD-DSRCT, SK-DSRCT1, SK-DSRCT2, and OV-054 DSRCT) that are enabling more robust DSRCT research (Bleijs et al., 2021; Smith et al., 2022). Recent efforts have also led to the creation of large tumor banks and the establishment of a variety of patient-derived xenografts (PDXs). PDX models established include at least one at Fondazione IRCSS Istituto Nazionale dei Tumori, three at MD Anderson Cancer Center, and a set of twenty-four at Memorial Sloan Kettering Cancer Center (Slotkin et al., 2021a; Zuco et al., 2023a; Truong et al., 2023). Memorial Sloan Kettering is one of the primary referral centers for DSRCT patients and has further established a tumor bank with >200 DSRCT samples.

These new resources have been coupled with next-generation sequencing technologies to establish critical datasets and insights. DNA sequencing studies have sought to identify recurrent mutations in DSRCT other than the EWSR1::WT1 fusion. Chow et al. performed targeted sequencing of 405 genes from 83 DSRCT tumors and found recurrent mutations in FGFR4 (8%), ARID1A (11%), TP53 (10%), MSH3 (14%), and MLL3 (16%) (Chow et al., 2020). Slotkin et al. performed targeted sequencing of 468 genes in 68 samples and full genome sequencing in 10 samples, identifying recurrent mutations in TERT (3%), ARID1A (6%), HRAS (5%), and TP53 (3%) (Slotkin et al., 2021a). Other studies with smaller tumor sets have similarly found a low tumor mutation burden and few recurrent mutations (Devecchi et al., 2018; Wu C. C. et al., 2022). In light of these findings, RNA sequencing (RNA-seq) may be more clinically informative, and a variety of studies are now sequencing DSRCT samples to understand the DSRCT transcriptome. Hingorani et al. and Wu et al. performed RNA-seq on 12 and 22 DSRCT tumors, respectively (Hingorani et al., 2020; Wu C. C. et al., 2022). More recently, we sequenced a set of 57 DSRCT tumors from the Memorial Sloan Kettering DSRCT Tumor Bank which will soon be made publicly available (unpublished). Three studies have performed RNA-seq on a total of 5 different DSRCT cell lines with and without depletion of EWSR1::WT1 (Gedminas et al., 2020; Bleijs et al., 2021; Magrath et al., 2024a). EWSR1::WT1 has two isoforms (E+KTS, E-KTS) generated from alternative splicing that either includes or excludes the amino acids lysine, threonine, and serine (abbreviated: KTS) between zinc fingers 3 and 4 of WT1 (Kim et al., 1998). Two studies have performed RNA-seq on mesothelial cell lines with overexpression of one or both of these two isoforms (Magrath et al., 2024a). Chromatin Immunoprecipitation and sequencing (ChIP-Seq) directed toward the fusion protein has been performed twice to identify EWSR1::WT1 binding locations in JN-DSRCT-1 as well as isoform specific binding in the MeT-5A mesothelial cell line (Hingorani et al., 2020). ChIP-Seq datasets have further been established examining androgen receptor (AR) binding and characterizing the landscape of histone modifications (Lamhamedi-Cherradi et al., 2022). Together, these datasets have not only led to many novel findings but also provide a critical tool kit for future research.

## 3 EWSR1::WT1 oncogenic mechanism

The EWSR1::WT1 oncogene is a transcription factor formed by the fusion of the N-terminal activating domain of EWSR1 with the final three zinc fingers at the C-terminus of WT1. The EWSR1 breakpoint can occur in one of three introns (Magrath et al., 2023a). However, the different variants induce many common transcriptomic alterations (Magrath et al., 2024a). The essentiality of EWSR1::WT1 has been established with shRNA knockdown *in vitro* in five independent studies (Kang et al., 2014; Gedminas et al., 2020; Bleijs et al., 2021; Hartono et al., 2022; Magrath et al., 2024a) across five DSRCT cell lines and more recently *in vivo* using doxycycline-inducible shRNA depletion of EWSR1::WT1 in 3 cell line-derived xenografts (Magrath et al., 2024a). EWSR1::WT1 is present in the nucleus and acts as a transcription factor, both binding to DNA and altering transcription (Gedminas et al., 2020; Hingorani et al., 2020). Recent studies have established sets of genes commonly regulated by EWSR1::WT1 across cell lines including a set of 68 upregulated and 223 downregulated genes identified across 2 cell lines by Gedminas et al., 2020 and a set of 175 upregulated and 166 downregulated genes identified across 4 cell lines in our lab (Magrath et al., 2024a). Integrating this gene expression data and WT1-directed ChIP-seq, we found that EWSR1::WT1 binding predominantly leads to upregulation or stable gene expression, with only 10% of EWSR1::WT1 bound genes negatively regulated by the fusion protein (Magrath et al., 2024a). The native WT1 motif is enriched at EWSR1::WT1 bound sites. However, the binding profile of EWSR1::WT1 in DSRCT differs significantly from WT1 in leukemia cells (Magrath et al., 2024a). This could be due to differences in cell type/epigenetic state or binding specificity and should be investigated in future work.

A unique aspect of EWSR1::WT1 is its existence in two isoforms (E + KTS and E-KTS) which vary based on the inclusion or exclusion of three amino acids between zinc fingers 3 and 4 of the WT1 DNA binding domain. These isoform variants also occur in native WT1, where the difference in distance between zinc fingers 3 and 4 leads to differences in DNA binding and transcription (Potluri et al., 2021). In EWSR1::WT1, the isoforms were initially studied in mouse models where they led to differences in transcription, with most alterations caused by the E-KTS isoform including upregulation of pathways related to neuronal reprogramming (Kang et al., 2014). These findings have been validated and expanded in human cell lines where most upregulation and downregulation is caused by the E-KTS isoform including upregulation of critical proliferation signals such as CCND1 (Magrath et al., 2024a). Given the differences in isoform transcriptional activity, an important question in DSRCT biology is whether one, the other, or both isoforms are necessary to induce oncogenesis. Kim et al. found that E-KTS but not E + KTS was able to increase the growth rate and lead to tumor formation in NIH3T3 mouse fibroblast cells (Kim et al., 1998). In contrast, Bandopadhayay et al., 2013. found that neither isoform was able to transform p53 wildtype murine embryonic fibroblasts, while either isoform led to increased colony formation in p53 mutant murine embryonic fibroblasts. This suggests p53 mutations may be important for EWSR1::WT1 induced oncogenesis. However, DNA sequencing studies have consistently found few p53 mutations in DSRCT (Slotkin et al., 2021a). In our own hands, we find that transduction of normal human mesothelial cell line LP9 with one or both isoforms is insufficient to induce transformation and that E-KTS expression decreases rather than increases growth, perhaps due to an oncogenic stress response (Haigis and Sweet-Cordero, 2011). These data strongly suggest additional alterations or epigenetic states are necessary for EWSR1::WT1 induced oncogenesis. It may be that p53 mutation is one of several potential prerequisite alterations that can prime cells for EWSR1::WT1 induced transformation. Both the role of these isoforms and their interaction with other mutations require further research. Other mechanistic questions that require further investigation include the role of EWSR1::WT1 in altering the chromatin landscape and whether it has oncogenic functions independent of its role as a transcription factor. While the recent availability of new tools for investigating DSRCT has led to many advances in our understanding, the mechanistic framework lags behind other fusion oncoprotein driven tumors such as Ewing sarcoma and necessitates further study.

## 4 Promising targeted therapeutic strategies

Given DSRCT’s extremely poor prognosis, novel targeted therapies are urgently needed. The recent development of DSRCT models has enabled a rapid expansion in preclinical investigation and the proposal of many novel therapeutic targets over the past 5 years. These proposals have focused on two primary strategies: 1) inhibition of critical growth pathways and 2) immunotherapy directed toward highly expressed surface proteins. Tables 1, 2 summarize the candidate targets identified and investigated preclinically using these two strategies. Table 3 summarizes ongoing clinical trials for DSRCT examining these targets. While most therapeutic targets have been identified due to their role as downstream targets of EWSR1::WT1, a few therapeutic targets are not regulated by EWSR1::WT1 and were instead identified due to their high expression in DSRCT tumors. In contrast to other pediatric cancers such as Ewing sarcoma, functional screening to comprehensively identify critical dependencies in DSRCT has not yet been established. Application of this technology to DSRCT should be prioritized in future studies which can utilize CRISPR knockout screening to not only identify DSRCT critical dependencies but also select targets that uniquely regulate EWSR1::WT1 expression. This application will likely be a critical source for the next set of DSRCT therapeutic targets. The results of this screening can be further combined with single cell sequencing technologies that will provide insights on tumor heterogeneity. Together current therapeutic targets and the novel targets and insights gained from these technologies can enable the establishment of clinically relevant treatments. Here we examine the most promising findings to date and their potential for clinical translation.

**TABLE 1:** *DSRCT Inhibitor Targets*.

Target	EWSR1::WT1 regulation	Drug availability	Xenograft results	Reference
NTRK3	Downstream	Entrectinib*, Repotrectinib, PBI-200	Entrectinib treatment reduced growth in 3 subcutaneous patient-derived xenografts	Ogura et al. (2021)
SIK1	Downstream	YKL-05-099 (pan-SIKi)	SIK1 depletion with shRNA reduced growth in 2 cell line derived subcutaneous xenografts	Hartono et al. (2022)
BLK	Downstream	Dasatinib*, PP2	Dasatinib treatment did not reduce growth of JN-DSRCT-1 subcutaneous xenografts	van Erp et al. (2022), Magrath et al. (2024b)
CCND-CDK4/6-RB Axis	Downstream	Palbociclib*, Abemaciclib*	Palbociclib reduced growth in 2 cell line derived subcutaneous xenografts	Magrath et al. (2024a)
EGFR	No	Cetuximab*, Afatinib*	Combination of cetuximab and afatinib led to reduced growth in 2 cell line derived subcutaneous xenografts and one patient-derived xenograft	Smith et al. (2022)
AR	No	Enzalutamide*, Flutamide*, Darolutamide	Enzalutamide or AR-directed anti-sense oligonucleotide reduced DSRCT growth in 1 cell line derived and one patient derived xenograft model	Lamhamedi-Cherradi et al. (2022)
PARP	No	Olaparib*	Combination of Olaparib and temozolomide significantly reduced tumor growth in JN-DSRCT-1 derived subcutaneous xenografts	van Erp et al. (2020)
Unknown target of Lurbinectedin	N/A	Lurbinectedin*, Trabectedin*	Lurbinectedin reduced growth in 1 cell line derived subcutaneous xenograft and one subcutaneous PDX.	Gedminas et al. (2022b)
CHK1	No	Prexasertib	Prexasertib led to complete regression of two subcutaneous PDX.	Lowery et al. (2019)

^*^Indicates the drug has been approved by the FDA.

**TABLE 2:** *DSRCT Immunotherapy Targets*.

Target	EWSR1::WT1 regulation	Established antibody	Xenograft results	Reference
B7-H3	No	8H9	None; But tested in clinical trials using various immunotherapy modalities	Espinosa-Cotton et al. (2023)
ERBB2/HER2	No	Traztuzumab*	Bispecific antibody treatment led to tumor regression in 2 cell line derived orthotopic xenograft models	Espinosa-Cotton et al. (2023)
EGFR	No	Cetuximab*	Bispecific antibody treatment did not reduce tumor growth in 1 cell line derived orthotopic xenograft model	Espinosa-Cotton et al. (2023)
Mesothelin	No	MORAb-009	Bispecific antibody treatment did not reduce tumor growth in 1 cell line derived orthotopic xenograft model	Espinosa-Cotton et al. (2023)
EWSR1::WT1 Breakpoint	N/A	None	None	Banks et al. (2023)
Neogenes	Downstream	None	None	Vibert et al. (2022), Truong et al. (2024)

^*^Indicates the drug has been approved by the FDA.

**TABLE 3:** *Active clinical trials for DSRCT patients*.

Trial number	Phase, status	Treatment	Sponsor	Disease indications
NCT04901806	Phase I/II, active, not recruiting	PBI-200	Pyramid Biosciences	Solid Tumor, Adult Brain Tumor, Primary DSRCT
NCT05918640	Phase I/II, recruiting	Lurbinectedin	Children’s Hospital of Philadelphia	FET-fused tumors, Ewing sarcoma, DSRCT, undifferentiated sarcoma
NCT02982486	Phase II, unknown	Nivolumab plus ipilimumab	Assaf-Harofeh Medical Center	Non-resectable sarcoma and endometrial carcinoma
NCT04483778	Phase I, active, not recruiting	B7-H3 CAR T cells	Seattle Children’s Hospital	Recurrent/refractory solid tumors in children and young adults
NCT04897321	Phase I, recruiting	B7-H3 CAR T cells	St. Jude Children’s Research Hospital	Pediatric patients with solid tumors
NCT04022213	Phase II, recruiting	Intraperitoneal RIT with^131^I-Omburtamab (B7-H3 Ab)	Memorial Sloan Kettering Cancer Center	Patients with DSRCT and other solid tumors involving the peritoneum

### 4.1 Inhibition of critical growth pathways

#### 4.1.1 NTRK3

Neurotrophic receptor tyrosine kinase 3 (NTRK3) is a transmembrane receptor tyrosine kinase involved in nervous system development and function. It is one of the three members of the NTRK family (NTRK1, NTRK2, NTRK3), all of which have been identified in fusion oncoproteins such as ETV6::NTRK3 which drives secretory breast cancer and infantile fibrosarcoma (Cocco et al., 2018). Ogura et al. identified NTRK3 as 1 of 1501 human promoters bound by EWSR1::WT1 in a ChIP chip array in 2 cell lines (Ogura et al., 2021). Transcriptomic analysis of EWSR1::WT1 regulated genes and the existence of entrectinib as an FDA approved therapy targeting NTRK3 further narrowed their focus (Table 1). Entrectinib treatment reduced the viability of two DSRCT cell lines (JN-DSRCT-1, SK-DSRCT2) *in vitro* and decreased phosphorylation of the mitogenic mediator ERK (Ogura et al., 2021). The role of NTRK3 in DSRCT was confirmed genetically via depletion with two independent shRNAs which led to reduced cell viability in both DSRCT cell lines. Examination of entrectinib efficacy in three patient-derived xenografts found near complete growth cessation in one xenograft and growth reductions of 50% and 75% in the other two xenografts (Ogura et al., 2021). Based on these results, DSRCT has been included in an ongoing clinical trial testing the efficacy of the next-generation NTRK inhibitor PBI-200 on solid tumors (Table 3).

#### 4.1.2 SIK1

Salt-Inducible-Kinases (SIKs) are a member of the AMP-activated protein kinase (AMPK) family and known for their role in mediating metabolic homeostasis (Darling and Cohen, 2021). SIK1 is regulated by metabolic signaling and typically expressed in the adrenal cortex, neural, and adipose tissues, while SIK2 and SIK3 are more widely and constitutively expressed in many tissues (Sakamoto et al., 2018). SIK2 has a role in tumorigenesis in prostate and ovarian cancers, and SIK2 and SIK3 promote the growth of acute myeloid leukemia (AML) by inhibiting HDAC4 function (Bon et al., 2015; Tarumoto et al., 2018; Gao et al., 2020). Recent studies have indicated SIK1 acts as a tumor suppressor in many cancer types by promoting p53-dependent anoikis in breast cancer, inhibiting lipid metabolism reprogramming in pancreatic tumorigenesis, and mediating the tumor suppressor function of LKB1 in lung cancer (Cheng et al., 2009; Patra et al., 2018; Hollstein et al., 2019).

SIK1 was identified as a potential target due to its high expression in DSRCT compared to other sarcomas and its regulation by EWSR1::WT1. In contrast to other cancer types where SIK1 acts as a tumor suppressor, Hartono et al. found that SIK1 depletion with siRNA or shRNA reduced the viability of two DSRCT cell lines, indicating an oncogenic role for SIK1 in DSRCT (Hartono et al., 2022). Mechanistically, SIK1 depletion reduced phosphorylation of MCM2 at S27 and S41 leading to a block in DNA replication. Consistent with this, growth of xenografts derived from JN- and BER-DSRCT-shSIK1 cells was significantly reduced with SIK1 depletion. These findings suggest SIK1 as a promising DSRCT therapeutic target. However, no specific SIK1 small molecule inhibitor exists, serving as a barrier to rapid clinical translation. The pan-SIK inhibitor YKL-05-099 induced cytotoxicity in BER-DSRCT but not JN-DSRCT-1 when compared to the LP9 mesothelial cell line which served as a normal control (Hartono et al., 2022). The lack of sensitivity in JN-DSRCT-1 may be explained by the lack of specificity of this inhibitor and suggest the development of a SIK1 specific inhibitor as an urgent priority to help translate these preclinical findings to DSRCT patients.

#### 4.1.3 BLK

B-lymphocyte kinase (BLK) is expressed in B cells where it contributes to their activation and proliferation (Dymecki et al., 1990; Saouaf et al., 1994; Malek et al., 1998). In oncology, studies have suggested BLK as an oncogene in B and T cell lymphomas (Malek et al., 1998; Krejsgaard et al., 2009; Petersen et al., 2014; Petersen et al., 2017). Intriguingly, studies in a *Blk* knockout mouse demonstrated that B cell development and the humoral immune response are not substantially impacted by BLK depletion, suggesting BLK-specific inhibitors may have few on-target off-tumor effects (Texido et al., 2000). We identified BLK as a kinase upregulated not only by EWSR1::WT1, but also in DSRCT cancer stem cell-like (CSC) culture conditions (Magrath et al., 2024b). Under CSC culture conditions, DSRCT cells form tumorspheres, have increased SOX2 and NANOG expression, and are more resistant to chemotherapy, making it a potentially valuable tool for identifying therapies that can target chemoresistant DSRCT cells (Magrath et al., 2022). Treatment with dasatinib and PP2, which inhibit BLK as well as other SRC family members, decreased DSRCT sphere formation, CSC culture viability, and stemness marker expression. Depletion of BLK with shRNA similarly reduced tumorsphere formation and stemness marker expression, and also sensitized DSRCT CSCs to doxorubicin treatment (Magrath et al., 2024b). While BLK inhibition can reduce CSC characteristics, BLK is not expressed highly in bulk tumor cells and a study by Van Erp et al., 2022 *.* found that dasatinib monotherapy failed to reduced growth of JN-DSRCT-1 xenografts. Together, these findings suggest BLK inhibition alone will be an ineffective DSRCT therapeutic but combination of BLK inhibition with other therapies targeting bulk tumor cells is worthy of further examination.

#### 4.1.4 CCND-CDK4/6-RB axis

The CCND-CDK4/6-RB axis is a critical regulator of the transition from G1 to S phase of the cell cycle and is dysregulated in a variety of cancers (VanArsdale et al., 2015). In combination with estrogen receptor inhibition, CDK4/6 inhibitors (palbociclib, abemaciclib, ribociclib) prolong survival in breast cancer and have led to a new therapeutic paradigm (Cristofanilli et al., 2016; Turner et al., 2018). CDK4/6 inhibitors are also effective against liposarcoma (Hsu et al., 2022) clinically and have demonstrated preclinical efficacy in Ewing sarcoma (Kennedy et al., 2015) and neuroblastoma (Rader et al., 2013). We identified CDK4/6 inhibitors as a promising therapeutic for DSRCT due to the control of this axis by EWSR1::WT1 (Magrath et al., 2024a). The E-KTS isoform binds to and regulates expression of CCND1, which in turn activates CDK4/6, enabling DSRCT cells to overcome the RB tumor suppressor and proliferate. Palbociclib treatment effectively reduced RB phosphorylation and significantly decreased tumor growth in 2 cell line-derived xenografts (Magrath et al., 2024a). The primary mechanism of CDK4/6i resistance in tumor is RB inactivation. However, genomic studies of DSRCT (Slotkin et al., 2021a; Wu C.-C. et al., 2022) have not identified frequent RB mutations, making it likely that most DSRCT tumors will be at least initially responsive to CDK4/6 inhibition.

#### 4.1.5 EGFR

Epidermal growth factor receptor (EGFR) is a receptor tyrosine kinase (RTK) which plays an important role in cell division and growth in epithelial cells (Talukdar et al., 2020). EGFR is overexpressed in glioblastoma, breast, and lung cancers leading to dysregulation of the EGFR pathway and increased cell proliferation (Sigismund et al., 2018; Talukdar et al., 2020; Uribe et al., 2021). EGFR was identified in DSRCT by Smith et al. due to the enrichment of EGFR-related pathways in a gene set variation analysis of DSRCT microarray data (Smith et al., 2022). While EGFR is highly expressed in DSRCT, it is not transcriptionally regulated by EWSR1::WT1, in contrast to other proposed therapeutic targets to date. Smith et al. demonstrated through both chemical inhibition and genetic silencing that EGFR is critical to DSRCT growth. Multiple EGFR-targeting therapeutics including afatinib, neratinib, and cetuximab are FDA approved for the treatment of other cancers and could be quickly applied to DSRCT in the clinic, making EGFR an exciting therapeutic target. In mice, cetuximab monotherapy or combination therapy with cetuximab and afatinib reduced tumor growth in 2 cell line derived xenografts, while afatinib therapy alone had a limited effect. In a PDX model, the combination therapy of cetuximab and afatinib led to a 40% reduction in tumor volume over a 49-day treatment (Smith et al., 2022). While likely insufficient as a monotherapy, these data suggest EGFR targeting can reduce DSRCT growth *in vivo* and could be combined with other therapies to produce a greater therapeutic response. The success of targeting EGFR in combination therapy may be better understood through further elucidation of its relationship with EWSR1::WT1. Smith et al. found that several ligands of the ERBB system were upregulated in LP9 cells with exogenous expression of EWSR1::WT1 including *EGF*, *NEUREGULIN 1* (*NRG1*), *EPIREGULIN* (*EPGN*), and *AMPHIREGULIN* (*AREG*). This could suggest EWSR1::WT1 regulation of these targets drives EGFR-dependent growth in DSRCT and that this pathway is in fact fusion protein regulated. However, more recent RNA-seq data with EWSR1::WT1 depletion has not found consistent downregulation of these ligands (Magrath et al., 2024a). The role of these ligands in DSRCT survival also remains uninvestigated. If EGFR is determined to be an EWSR1::WT1-independent DSRCT dependency, this could allow combined therapy targeting EWSR1::WT1 dependent and independent pathways which may be necessary to improve prognosis.

#### 4.1.6 AR

DSRCT’s high male predominance has led to several investigations to uncover a potential role of the androgen receptor (AR) in its pathogenesis. Similar to EGFR, AR expression is not transcriptionally controlled by EWSR1::WT1 and represents a potential EWSR1::WT1 independent critical pathway (Magrath et al., 2023b). In 2007, Fine et al., 2007 identified AR positivity in immunohistochemistry samples from 10 of 27 DSRCT patients and discovered that flutamide treatment reduced DSRCT growth *in vitro*. Translating these findings to the clinic, they found three out of six DSRCT patients responded to combined androgen blockade (bicalutamide and Lupron), with two patients experiencing a partial response for 3 months and a third patient experiencing stable disease (Fine et al., 2007). In 2022, Lamhamedi-Cherradi et al., 2022 expanded on these findings and suggested a higher rate of AR positivity in DSRCT of 75%. Testing 1 cell line derived and one patient derived xenograft, Lamhamedi-Cherradi et al., 2022 *.* found that treatment with either enzalutamide or an AR-directed antisense oligonucleotide substantially reduced tumor growth *in vivo*. Similarly, we began investigating AR as a result of its male predominance and showed that three DSRCT cell lines respond to high dose treatment with enzalutamide, flutamide, and darolutamide (Magrath et al., 2024c). Using immunoprecipitation, we identified an interaction between AR and EWSR1::WT1 which may explain some of their shared binding locations in the genome. However, despite these findings suggesting an important role of AR in DSRCT, we found that knockdown of AR in two DSRCT cell lines (JN-DSRCT-1, BER-DSRCT) with four independent shRNAs failed to reduce DSRCT viability and that enzalutamide and flutamide cytotoxicity remained after AR depletion (Magrath et al., 2024c). These findings suggest the AR antagonists may act through an AR-independent mechanism in DSRCT which may involve other members of the NR3 nuclear receptor pathway such as the glucocorticoid receptor which is highly expressed in DSRCT (Magrath et al., 2024c). Intriguingly, we found that enzalutamide treatment reduced EWSR1::WT1 expression which could explain its efficacy. Further research is necessary to better understand the mechanism of AR antagonists and their potential role in suppression of EWSR1::WT1. As many AR directed therapies are FDA approved for the treatment of prostate cancer, better elucidation of their efficacy and mechanism in DSRCT could lead to quick clinical translation. Even though AR may not be one of the drivers of DSRCT, AR could still play a pivotal role in the initiation of DSRCT. Previous studies showed that AR binds to genomic regions of TMPRSS2 and ERG and facilitates chromosomal translocation in prostate cancers (Nicholas et al., 2021). AR has been shown to bind to introns of EWSR1 and WT1 (Lamhamedi-Cherradi et al., 2022; Magrath et al., 2024c), raising a tantalizing possibility that similar AR-induced chromosomal translocation could be responsible for generating the pathognomonic alteration and the male predominance in DSRCT.

#### 4.1.7 PARP

Poly-ADP ribose polymerase (PARP) belongs to a family of proteins involved in various cellular processes including DNA transcription, replication, and repair, programmed cell death, chromatin structure modulation, and genomic stability. PARP enzymes function by catalyzing the transfer of ADP-ribose to target proteins (Amé et al., 2004). PARP is overexpressed in different types of cancers and its inhibition using FDA approved drugs like olaparib, rucaparib, and veliparib are therapeutics for treating cancers of the breast, ovary, prostate, pancreas, and other solid tumors (Wang et al., 2017). During DNA damage, PARP binds to damaged DNA and recruits DNA repair proteins to the site of damage leading to DNA repair. PARP inhibitors trap PARP within the damaged DNA leading to damage accumulation. Combination of PARP inhibitors with DNA damaging agents is often synergistic and can improve lethality. In DSRCT, PARP was identified as a potential therapeutic target due to its high expression in 16/16 DSRCT IHC samples (van Erp et al., 2020). As a monotherapy, the PARP inhibitor olaparib reduced the viability of JN-DSRCT-1 cells with an IC_50_ of 1.38 µM^72^. Intriguingly, the combination of olaparib with temozolomide induced synergistic reductions in viability and increased apoptosis. In a JN-DSRCT-1 xenograft model, neither olaparib nor temozolomide monotherapy significantly reduced tumor growth. However, in combination, a significant growth reduction was seen (van Erp et al., 2020). This data demonstrates the value of PARP inhibition with other DNA damaging agents in DSRCT which warrants investigation in other DSRCT models.

#### 4.1.8 Unknown target of trabectedin/lurbinectedin

Trabectedin and lurbinectedin are alkylating agents that interact with the minor groove of DNA and many DNA binding proteins (Grosso et al., 2007; Grosso et al., 2009; Di Giandomenico et al., 2014; Kauffmann-Guerrero and Huber, 2020). Trabectedin is a naturally occurring compound while lurbinectedin is a synthetic variant of trabectedin where tetrahydro β‐carboline is substituted for tetrahydroisoquinoline to improve pharmacokinetics and reduce toxicity (Kauffmann-Guerrero and Huber, 2020). Both compounds induce DNA damage and disrupt transcription. This latter mechanism has led to investigations into their ability to treat transcriptionally driven cancers such as those caused by fusion oncoproteins. Trabectedin decreases the DNA binding of the FUS::CHOP oncogenic transcription factor in myxoid liposarcoma and has led to both partial and complete clinical responses (Grosso et al., 2007; Grosso et al., 2009; Di Giandomenico et al., 2014). Trabectedin has additionally shown efficacy in the treatment of liposarcoma, leiomyosarcoma, and ovarian cancer (Demetri et al., 2016; Teplinsky and Herzog, 2017). Similarly, lurbinectedin has shown preclinical effects on EWSR1::FLI1 in Ewing sarcoma (Harlow et al., 2016).

Trabectedin was first investigated preclinically in DSRCT by Uboldi et al. who showed that JN-DSRCT-1 cells are sensitive to trabectedin with an IC_50_ around 4.5 nM^80^. Using Chromatin Immunoprecipitation assays, they found that trabectedin treatment significantly reduced EWSR1::WT1 binding to the promoter of three genes highly expressed in DSRCT (Uboldi et al., 2017). Gedminas et al. extended these findings to lurbinectedin, finding that exposing DSRCT cells to lurbinectedin decreased growth and led to repression of 80% of EWSR1::WT1 regulated targets (Gedminas et al., 2022b). Intriguingly, they showed that lurbinectedin treatment led to localization of fusion protein to the nucleolus and reduced overall EWSR1::WT1 protein expression. *In vivo*, lurbinectedin led to significant reductions in tumor growth in 1 cell line-derived and one patient-derived xenograft (Gedminas et al., 2022b). Most recently, Zuco et al. utilized a novel DSRCT PDX model to examine the anticancer effectiveness of several chemotherapy agents including trabectedin (Zuco et al., 2023b). Individually, trabectedin had a maximum tumor volume inhibition of 82% while irinotecan led to a >99% response. However, discontinuation of either monotherapy led to a steep increase in tumor volume. In contrast, combination treatment with irinotecan and trabectedin resulted in a complete and sustained response for 86 days after therapy discontinuation (Zuco et al., 2023b). RNA-seq analysis of treated tumors found that the irinotecan-trabectedin combination treatment, but not either monotherapy, resulted in downregulation of E2F targets, G2M checkpoint, and mitotic spindle gene sets (Zuco et al., 2023b). Intriguingly, combination therapy but not trabectedin monotherapy also led to reductions in the EWSR1::WT1 regulated targets NTRK3 and EGR1 at the protein and gene transcription levels (Zuco et al., 2023b). This suggests trabectedin alone may act through a mechanism other than EWSR1::WT1 depletion, which is in contrast to the proposed mechanism of lurbinectedin by Gedminas et al. Given the similarity of these compounds, further investigation is required to understand the true mechanism of action of lurbinectedin and trabectedin, whether they are the same, and whether they are true inhibitors of EWSR1::WT1. Regardless of their mechanism, lurbinectedin and trabectedin stand as intriguing therapeutics for DSRCT and have led to a few notable responses in case studies. In a report of two patients treated with the combination of trabectedin and irinotecan, one patient experienced stable disease while the other experienced a complete remission (Ferrari et al., 2023). There is currently an ongoing Phase I/II clinical trial examining the potential of lurbinectedin therapy for the treatment of FET-fused tumors including Ewing sarcoma and DSRCT (Table 3).

#### 4.1.9 CHK1

Checkpoint kinase 1 (CHK1) is a serine/threonine kinase involved in the DNA damage response, specifically homologous repair of double strand breaks. Activation of CHK1 leads to cell cycle arrest in the G2/M phase and eventual cell death due to mitotic catastrophe (Sanchez et al., 1997). As DSRCT is known to upregulate expression of DNA damage response elements, it stands to reason that interfering with this dependency would negatively impact tumor cell survival (Gedminas et al., 2020). Indeed, in a study of 38 pediatric sarcoma CDX and PDX models treated with prexasertib monotherapy, the two DSRCT PDX included in the study exhibited complete regression (Lowery et al., 2019). In one PDX, the initial response to prexasertib was durable. The other recurred 2 months after treatment but remained sensitive to prexasertib and following retreatment did not recur. A Phase I/II clincal trial of prexasertib + irinotecan was conducted with DSRCT and rhabdomyosarcoma patients (19 and 2 patients, respectively) with relapsed or refractory disease. The study met its primary objective to consider this drug combination for further investigation in DSRCT, with 32% of patients achieving partial response and 47% of patients achieving stable disease (Slotkin et al., 2022). Notably, the majority of patients who responded had previously been treated with irinotecan, suggesting that combination with prexasertib may resensitize tumors to irinotecan. Development of prexasertib was discontinued by Eli Lilly in 2019, but the drug was later licensed by Acrivon Therapeutics, allowing for the possibility of its availability for future trials.

### 4.2 Immunotherapy, radioimmunotherapy, and antibody-drug conjugates

Antibody-based therapeutics have the potential to deliver cytotoxic immune cells (monoclonal antibodies [mAbs] and T cell-engaging bispecific antibodies [T-BsAbs]), radionuclides (radioimmunotherapy [RIT]), and chemotherapeutic drugs (antibody-drug conjugates [ADC]) with specificity for tumors by targeting cell surface antigens expressed uniquely by or at high levels on the cell surface of cancer cells (Larson et al., 2015; Goydel and Rader, 2021). All of these can be delivered as off-the-shelf drugs. Chimeric antigen receptor (CAR) T cells also direct cytotoxic immune cells to tumors through targeting of cell surface tumor antigens, though in this strategy the T cells themselves are harvested, genetically engineered *ex vivo* to express receptors specific for these antigens, expanded, and reinfused into the patient (Sterner and Sterner, 2021). All these strategies face issues related to effectively delivering immune cells and large molecules (antibodies >150 kDa) within a tumor comprised of chaotic vasculature and a stiff, fibrotic extra-cellular matrix. DSRCT, as its name suggests, is densely stromal, similar in some ways to pancreatic ductal adenocarcinoma, protected by what has been described as a “fibrotic fortress” (Myo Min et al., 2023). This tumor microenvironment (TME) rich in cancer-associated fibroblasts (CAFs) results in not only a physical barrier to drug diffusion and immune cell trafficking, but also promotes immunosuppression through the secretion of chemokines and cytokines that induce differentiation of regulatory T cells and myeloid-derived suppressive cells (Monteran and Erez, 2019). Terry et al. describe various strategies to overcome these challenges specifically in the context of CAR T cells for pediatric sarcomas (Terry et al., 2021), but any of these could be employed to improve efficacy of other immunotherapy strategies as well. Immune checkpoint inhibitors (ICIs), which disrupt immunosuppressive interactions between T cells and tumor cells, rely on both the presence of sufficient numbers of immune cells in the TME and the existence of tumor neoantigens, both of which are commonly lacking in pediatric sarcomas with relatively silent genomes such as DSRCT (Negri et al., 2017; Slotkin et al., 2021b; Wu C. C. et al., 2022; Shiravand et al., 2022; Anzar et al., 2023). While ICIs have led to significant benefits for adult cancers with high mutational burden, they have largely failed in clinical trials for adolescents and young adults with low-mutational burden sarcomas (Tawbi et al., 2017; Blay et al., 2023), although anecdotal reports of partial responses in DSRCT patients do exist (Schöffski et al., 2023). While it is plausible that this class of drugs may synergize with other immunotherapy strategies (T-BsAbs, CAR T cells), as monotherapy they are not promising for DSRCT in the absence of neoantigens and a robust immune cell population in the TME. Recent work on immunotherapy in DSRCT has therefore focused on identifying and developing new targets for antibody-based treatments including 1) testing previously identified DSRCT immunotherapy targets such as B7-H3 in clinical trials, 2) investigating repurposed immunotherapy targets from other cancer types in DSRCT xenograft models, and 3) identifying new, more specific DSRCT immunotherapy targets.

#### 4.2.1 B7-H3

B7 homolog 3 (B7-H3) was the first target antigen identified in DSRCT and since then has been the most well-validated in the clinic (Modak et al., 2002). Its strong, homogenous, and nearly ubiquitous expression in DSRCT makes it an excellent candidate for antibody-based anti-cancer strategies (Modak et al., 2002; Espinosa-Cotton et al., 2023). A Phase I trial of intraperitoneal ^131^I-omburtamab (a radioantibody targeting B7-H3) opened in 2010 and results indicated that the treatment was safe, well-tolerated, and led to prolonged abdominal progression-free survival (Modak et al., 2020). The follow up Phase II trial opened in 2019 (NCT04022213, Table 3) and is expected to be completed in 2024. If successful, this will represent the first new treatment for DSRCT in decades, and could go on to be tested in other types of B7-H3-expressing cancer that spread within the peritoneum, including colorectal and ovarian cancer (Kontos et al., 2021). One pitfall this therapy will face is the prospect of extra-abdominal relapse, which was noted in several patients in the Phase I trial (Modak et al., 2020). However, strategies to safely administer curative doses of radioimmunotherapy systemically by decreasing myelotoxicity and other normal tissue toxicity are being investigated, including the use of two and three-step platforms and alternative antibody formats (Santich et al., 2021). B7-H3 CAR T cells are in Phase I trials for children and young adults at two different institutions (NCT04483778 and NCT04897321, Table 3) though it is unknown if any DSRCT patients have or will be enrolled in these trials and no results have yet been reported. In 2016, Macrogenics began enrolling pediatric patients with B7-H3-expressing solid tumors in a Phase I clinical trial (NCT02982941) for its Fc-enhanced B7-H3 mAb (enoblituzumab), but aside from a 2017 abstract confirming that enrollment had begun and was to include DSRCT patients, no updates or results have been published as of April 2024 (Desantes et al., 2017).

#### 4.2.2 HER2

Human epidermal growth factor receptor 2 (HER2) is most well-known for its role in breast cancer, for which the anti-HER2 mAb trastuzumab was developed. Since then, HER2 expression has been reported in many other solid tumor types, including sarcomas such as osteosarcoma and DSRCT (Zhang et al., 2003; Tabak et al., 2018; Espinosa-Cotton et al., 2023; Zhu et al., 2023). Because it is a growth factor receptor, anti-HER2 antibody-based therapeutics can inhibit tumor cell growth by downregulating proliferative pathways as well by redirecting immune cells or delivering cytotoxic payloads (Smith et al., 2022). Our group recently investigated a set of 8 potential immunotherapy targets in DSRCT (B7-H3, c-Met, EGFR, GD2, HER2, L1CAM, mesothelin, and polysialic acid) (Espinosa-Cotton et al., 2023). T-BsAb against HER2, EGFR, and mesothelin showed promising results in cytotoxicity assays *in vitro* and were further investigated in a humanized immunodeficient mouse model of intraperitoneal DSRCT. While T-BsAb targeting EGFR and mesothelin failed to significantly reduce tumor growth, HER2-targeted T-BsAbs displayed potent anti-tumor activity, eliminating tumors derived from two different cell lines with durable responses (Espinosa-Cotton et al., 2023). These findings are bolstered by a 2015 report of a DSRCT patient treated with HER2 CAR T cells in a basket trial for sarcoma patients who had stable disease for over 14 months after the initial infusion (Ahmed et al., 2015). However, it should be noted that this patient’s tumors were localized only to the liver at the time of treatment and they received 8 additional infusions of CAR T cells during their period of disease stabilization. Still, this experience suggests a possibility for HER2 CAR T cells to be safely and repeatedly administered to DSRCT patients to maintain remission. While yet untested clinically in DSRCT, HER2 ADCs are likely to be investigated in the future owing to their success in other HER2-positive solid tumors and the high expression of HER2 in DSRCT. Although trastuzumab deruxtecan failed in a recent Phase I trial for osteosarcoma (OS) (Hingorani et al., 2022), several issues with patient selection in this trial should be noted (Nakano, 2023). Specifically, patients with as few as 10% HER2 positive tumor cells were included, and the type of drug included in the payload, a topoisomerase I inhibitor, has not been shown to be effective against OS. In DSRCT, the topoisomerase I inhibitor irinotecan is under clinical investigation in numerous combinations with promising results and careful trial design could ensure that only patients with strong HER2 expression are included (Espinosa-Cotton et al., 2023; Hovsepyan et al., 2023).

#### 4.2.3 Neopeptides as neoantigens

Another intriguing set of potential DSRCT immunotherapy targets are neopeptides. Fusion proteins are themselves neopeptides with the amino acid sequence at the breakpoint junction serving as a neoantigen that can be targeted by the immune system (Wang et al., 2021). Studies have shown that a variety of fusion protein breakpoints can be recognized by T cells including BCR::ABL in chronic myelogenous leukemia and SYT::SSX in synovial sarcoma (Yotnda et al., 1998; Sato et al., 2002). Yang et al. attributed a complete remission of head and neck squamous cell carcinoma in a patient treated with anti-PD1 to T cells targeting the DEK::AFF2 fusion protein (Yang et al., 2019). In DSRCT, the EWSR1::WT1 breakpoint fuses either EWSR1 exon 7, 9, or 10 to exon 8 of WT1. The most common fusion is between exon 7 of EWSR1 and exon 8 of WT1 which was seen in 43/47 samples in our recent RNA-seq dataset. Work is currently underway using this junction as a therapeutic target and has been presented at conferences but not yet published (Banks et al., 2023).

A second source of neopeptides in DSRCT are neogenes. Neogenes are new genes created by oncogenic transcription factors that lead to novel mRNA in genomic regions normally not transcribed. Vibert et al. identified neogenes induced by the EWSR1::FLI oncoprotein in Ewing sarcoma including a few which are translated into neopeptides (Vibert et al., 2022). Vibert et al. further identified neogenes in 17 additional fusion protein driven cancers, including 37 neogenes in DSRCT. Building on this work, we examined the expression of these neogenes in an independent set of 22 DSRCT samples, finding that most neogenes are consistently expressed in DSRCT tumors (Truong et al., 2024). We further explored the transcriptional control of these neogenes across four DSRCT cell lines and identified seven neogenes whose expression was decreased by at least 75% in all cell lines upon EWSR1::WT1 knockdown (Truong et al., 2024). In Ewing sarcoma, EWSR1::FLI1 driven neogenes have been shown to be translated to neopeptides that are displayed on MHC and can be therapeutically targeted, increasing the probability that a similar therapeutic modality may be developed in DSRCT.

### 4.3 Combination therapy

While many novel therapies have been tested and demonstrated benefits in preclinical models, almost all have failed to induce durable tumor remission, partly because of tumor heterogeneity, partly because of intrinsic tumor resistance to a single modality approach. Combination therapy will likely be necessary to achieve the desired clinical outcome in DSRCT and could be a productive area of future investigation both in preclinical models and clinical applications. Combination therapy may include multiple inhibitors, multiple immunotherapy targets, or a combination of growth pathway inhibition and immunotherapy. In addition to addressing target heterogeneity, combination therapy takes advantage of fundamental differences in mechanisms of cell killing. For example, immunotherapy is generally not S-phase dependent, a dependency necessary for many chemotherapy drugs, and small molecule targets are often cytostatic hence requiring continuous long term administration unlike CARTs which are living drugs. When drugs that do not share toxicity profiles are combined to achieve synergy, the sum is much more than the parts, when they can be safely administered. In breast cancer, simultaneous inhibition of the estrogen receptor and CDK4/6 leads to greater reduction in RB phosphorylation than monotherapy and has led to significant gains in survival (VanArsdale et al., 2015; Cristofanilli et al., 2016; Turner et al., 2018). A similar strategy in DSRCT could combine an EWSR1::WT1 upstream regulator (yet to be identified) with entrectinib to inhibit NTRK3 or palbociclib to inhibit the CCND-CDK4/6-RB axis. It may also be beneficial to simultaneously inhibit EWSR1::WT1 dependent and independent pathways: for example, combination of EGFR inhibition (EWSR1::WT1 independent) and inhibition of NTRK3 or the CCND-CDK4/6-RB axis (EWSR1::WT1 dependent).

Combining multiple immunotherapy targets may be beneficial to increase specificity or account for expression heterogeneity among tumor cells. Dual targeting antibodies (Huang et al., 2020; Long et al., 2024) combining specificities for HER2 and B7-H3, or HER2 and EGFR may be more specific than bispecific antibodies targeting HER2 alone and thereby reduce on-target off-tumor side effects. Instead of carrying both specificities on the same antibody, multi-specific T cells can be created *ex vivo* by simple arming of polyclonal T cells with multiple BsAb specific for individual targets before iv injection (Park et al., 2021; Park and Cheung, 2022). Single cell RNA sequencing, proteomics and surfaceomics on DSRCT samples in the future can shed light on the heterogeneity profile of DSRCT cells to inform optimal immune target combination.

Combining critical pathway inhibitors and immunotherapy targets is an intriguing approach, but one where further investigation is required to understand the potential ramifications. Inhibitors could affect not only the DSRCT cells but also immune cells and stromal cells, the latter of which make up a large portion of DSRCT tumors. This could lead to synergistic or antagonistic effects which will likely be inhibitor-specific. BRAF and CDK4/6 inhibitors have recently been shown to increase immune cell infiltration and cytotoxic T cell activity, leading to synergistic effects when combined with immune checkpoint inhibitors in melanoma and lung cancer models (Hooijkaas et al., 2012; Wilmott et al., 2012; Liu et al., 2013; Deng et al., 2018; Lelliott et al., 2022). As CDK4/6 inhibitors have demonstrated efficacy in DSRCT (Magrath et al., 2024a), it may be beneficial to combine them with effective immunotherapy approaches, such as HER2-directed bispecific antibodies. NTRK3 is a particularly interesting target for combining inhibition and immunotherapy as it is both a critical dependency of DSRCT amenable to inhibition and expressed on the cell surface, making it potentially targetable by immunotherapy. In HER2+ breast cancer, inhibition with lapatinib increases the expression of HER2 and improves the efficacy of immune system mediated cytotoxicity induced by the HER2-targeting antibody trastuzumab (Scaltriti et al., 2009). A similar approach could lead to synergy in DSRCT with inhibition of NTRK3 kinase activity combined with NTRK3 targeting bispecific antibodies or CAR T cells. Conversely, combination therapy with an EWSR1::WT1 upstream inhibitor and NTRK3-directed immunotherapy may be predicted to have antagonistic effects. By reducing EWSR1::WT1 expression, the inhibitor could reduce NTRK3 expression and thus the efficacy of NTRK3-directed immunotherapy. Future preclinical investigations should therefore focus on both expanding the pool of DSRCT targeted therapies and testing the potential of combination therapies for synergy or antagonism.

## 5 Clinical translation

The recent availability of new DSRCT samples and cell lines has led to a rapid advance in the understanding of DSRCT biology and the identification of a variety of new therapeutic targets. Several of these targets are associated with pharmaceuticals that have already undergone safety testing and been approved by the FDA for the treatment of another malignancy. These targets, including NTRK3, EGFR, and CDK4/6, can be quickly translated to the clinic and should be urgently investigated in DSRCT. Inhibitors to all three of these targets have demonstrated efficacy in cell line-derived and/or patient-derived xenograft models. NTRK3 inhibition demonstrated efficacy in the highest number of PDXs at three and an active study is now examining the potential of the NTRK3 inhibitor PBI-200 in a cohort that includes DSRCT patients (NCT04901806). As none of these therapies are likely to eliminate all DSRCT cells alone, trials with arms for both single and combination therapy may be the best approach moving forward and should be prioritized by sarcoma researchers.

## References

[B1] Ahmed N. Brawley V. S. Hegde M. Robertson C. Ghazi A. Gerken C. (2015). Human epidermal growth factor receptor 2 (HER2) -specific chimeric antigen receptor-modified T cells for the immunotherapy of HER2-positive sarcoma. J. Clin. Oncol. 33, 1688–1696. 10.1200/jco.2014.58.0225 25800760 PMC4429176

[B2] Amé J. C. Spenlehauer C. de Murcia G. (2004). The PARP superfamily. Bioessays 26, 882–893. 10.1002/bies.20085 15273990

[B3] Anzar I. Malone B. Samarakoon P. Vardaxis I. Simovski B. Fontenelle H. (2023). The interplay between neoantigens and immune cells in sarcomas treated with checkpoint inhibition. Front. Immunol. 14, 1226445. 10.3389/fimmu.2023.1226445 37799721 PMC10548483

[B4] Bandopadhayay P. Jabbour A. M. Riffkin C. Salmanidis M. Gordon L. Popovski D. (2013). The oncogenic properties of EWS/WT1 of desmoplastic small round cell tumors are unmasked by loss of p53 in murine embryonic fibroblasts. BMC Cancer 13, 585. 10.1186/1471-2407-13-585 24321497 PMC4029184

[B5] Banks L. Arkin H. Chandran S. S. Slotkin E. Shukla N. Morris L. (2023). 343 Defining and therapeutically targeting a fusion-derived public neoantigen in desmoplastic small round cell tumor using T-cell receptor gene therapy. J. Immunother. Cancer 11, A392. 10.1136/jitc-2023-SITC2023.0343

[B6] Blay J. Y. Chevret S. Le Cesne A. Brahmi M. Penel N. Cousin S. (2023). Pembrolizumab in patients with rare and ultra-rare sarcomas (AcSé Pembrolizumab): analysis of a subgroup from a non-randomised, open-label, phase 2, basket trial. Lancet Oncol. 24, 892–902. 10.1016/s1470-2045(23)00282-6 37429302

[B7] Bleijs M. Pleijte C. Engels S. Ringnalda F. Meyer-Wentrup F. van de Wetering M. (2021). EWSR1-WT1 target genes and therapeutic options identified in a novel DSRCT *in vitro* model. Cancers (Basel) 13, 6072. 10.3390/cancers13236072 34885181 PMC8657306

[B8] Bon H. Wadhwa K. Schreiner A. Osborne M. Carroll T. Ramos-Montoya A. (2015). Salt-inducible kinase 2 regulates mitotic progression and transcription in prostate cancer. Mol. Cancer Res. 13, 620–635. 10.1158/1541-7786.MCR-13-0182-T 25548099 PMC4383640

[B9] Bulbul A. Fahy B. N. Xiu J. Rashad S. Mustafa A. Husain H. (2017). Desmoplastic small round blue cell tumor: a review of treatment and potential therapeutic genomic alterations. Sarcoma 2017, 1278268. 10.1155/2017/1278268 29225486 PMC5687144

[B10] Chang F. (2006). Desmoplastic small round cell tumors: cytologic, histologic, and immunohistochemical features. Archives Pathology Laboratory Med. 130, 728–732. 10.5858/2006-130-728-dsrctc 16683894

[B11] Cheng H. Liu P. Wang Z. C. Zou L. Santiago S. Garbitt V. (2009). SIK1 couples LKB1 to p53-dependent anoikis and suppresses metastasis. Sci. Signal. 2, ra35. 10.1126/scisignal.2000369 19622832 PMC2752275

[B12] Chow W. A. Yee J. K. Tsark W. Wu X. Qin H. Guan M. (2020). Recurrent secondary genomic alterations in desmoplastic small round cell tumors. BMC Med. Genet. 21, 101. 10.1186/s12881-020-01034-w 32393201 PMC7216377

[B13] Cocco E. Scaltriti M. Drilon A. (2018). NTRK fusion-positive cancers and TRK inhibitor therapy. Nat. Rev. Clin. Oncol. 15, 731–747. 10.1038/s41571-018-0113-0 30333516 PMC6419506

[B14] Cristofanilli M. Turner N. C. Bondarenko I. Ro J. Im S. A. Masuda N. (2016). Fulvestrant plus palbociclib versus fulvestrant plus placebo for treatment of hormone-receptor-positive, HER2-negative metastatic breast cancer that progressed on previous endocrine therapy (PALOMA-3): final analysis of the multicentre, double-blind, phase 3 randomised controlled trial. Lancet Oncol. 17, 425–439. 10.1016/s1470-2045(15)00613-0 26947331

[B15] Darling N. J. Cohen P. (2021). Nuts and bolts of the salt-inducible kinases (SIKs). Biochem. J. 478, 1377–1397. 10.1042/BCJ20200502 33861845 PMC8057676

[B16] Demetri G. D. von Mehren M. Jones R. L. Hensley M. L. Schuetze S. M. Staddon A. (2016). Efficacy and safety of trabectedin or dacarbazine for metastatic liposarcoma or leiomyosarcoma after failure of conventional chemotherapy: results of a phase III randomized multicenter clinical trial. J. Clin. Oncol. 34, 786–793. 10.1200/JCO.2015.62.4734 26371143 PMC5070559

[B17] Deng J. Wang E. S. Jenkins R. W. Li S. Dries R. Yates K. (2018). CDK4/6 inhibition augments antitumor immunity by enhancing T-cell activation. Cancer Discov. 8, 216–233. 10.1158/2159-8290.Cd-17-0915 29101163 PMC5809273

[B18] Desantes K. Maris J. M. McDowell K. Mackall C. Shankar S. Vasselli J. (2017). A phase 1, open-label, dose escalation study of enoblituzumab (MGA271) in pediatric patients with B7-H3-expressing relapsed or refractory solid tumors. J. Clin. Oncol. 35, TPS2596. 10.1200/JCO.2017.35.15_suppl.TPS2596 PMC12421222 40794737

[B19] Devecchi A. De Cecco L. Dugo M. Penso D. Dagrada G. Brich S. (2018). The genomics of desmoplastic small round cell tumor reveals the deregulation of genes related to DNA damage response, epithelial–mesenchymal transition, and immune response. Cancer Commun. 38, 70. 10.1186/s40880-018-0339-3 PMC6260689 30486883

[B20] Di Giandomenico S. Frapolli R. Bello E. Uboldi S. Licandro S. A. Marchini S. (2014). Mode of action of trabectedin in myxoid liposarcomas. Oncogene 33, 5201–5210. 10.1038/onc.2013.462 24213580

[B21] Dymecki S. M. Niederhuber J. E. Desiderio S. V. (1990). Specific expression of a tyrosine kinase gene, blk, in B lymphoid cells. Science 247, 332–336. 10.1126/science.2404338 2404338

[B22] Espinosa-Cotton M. Guo H. F. Tickoo S. K. Cheung N. V. (2023). Identification of immunotherapy and radioimmunotherapy targets on desmoplastic small round cell tumors. Front. Oncol. 13, 1104693. 10.3389/fonc.2023.1104693 37091153 PMC10119788

[B23] Ferrari A. Chiaravalli S. Bergamaschi L. Nigro O. Livellara V. Sironi G. (2023). Trabectedin-irinotecan, a potentially promising combination in relapsed desmoplastic small round cell tumor: report of two cases. J. Chemother. 35, 163–167. 10.1080/1120009x.2022.2067706 35470779

[B24] Fine R. L. Shah S. S. Moulton T. A. Yu I. R. Fogelman D. R. Richardson M. (2007). Androgen and c-Kit receptors in desmoplastic small round cell tumors resistant to chemotherapy: novel targets for therapy. Cancer Chemother. Pharmacol. 59, 429–437. 10.1007/s00280-006-0280-z 16896931

[B25] Gao T. Zhang X. Zhao J. Zhou F. Wang Y. Zhao Z. (2020). SIK2 promotes reprogramming of glucose metabolism through PI3K/AKT/HIF-1α pathway and Drp1-mediated mitochondrial fission in ovarian cancer. Cancer Lett. 469, 89–101. 10.1016/j.canlet.2019.10.029 31639424

[B26] Gedminas J. M. Chasse M. H. McBrairty M. Beddows I. Kitchen-Goosen S. M. Grohar P. J. (2020). Desmoplastic small round cell tumor is dependent on the EWS-WT1 transcription factor. Oncogenesis 9, 41. 10.1038/s41389-020-0224-1 32345977 PMC7188842

[B27] Gedminas J. M. Kaufman R. Boguslawski E. A. Gross A. C. Adams M. Beddows I. (2022a). Lurbinectedin inhibits the EWS-WT1 transcription factor in desmoplastic small round cell tumor. Mol. Cancer Ther. 21, 1296–1305. 10.1158/1535-7163.Mct-21-1003 35657345

[B28] Gedminas J. M. Kaufman R. Boguslawski E. A. Gross A. C. Adams M. Beddows I. (2022b). Lurbinectedin inhibits the EWS–WT1 transcription factor in desmoplastic small round cell tumor. Mol. cancer Ther. 21, 1296–1305. 10.1158/1535-7163.MCT-21-1003 35657345

[B29] Gerald W. L. Haber D. A. (2005). The EWS-WT1 gene fusion in desmoplastic small round cell tumor. Semin. Cancer Biol. 15, 197–205. 10.1016/j.semcancer.2005.01.005 15826834

[B30] Gerald W. L. Rosai J. Ladanyi M. (1995). Characterization of the genomic breakpoint and chimeric transcripts in the EWS-WT1 gene fusion of desmoplastic small round cell tumor. Proc. Natl. Acad. Sci. U. S. A. 92, 1028–1032. 10.1073/pnas.92.4.1028 7862627 PMC42630

[B31] Goydel R. S. Rader C. (2021). Antibody-based cancer therapy. Oncogene 40, 3655–3664. 10.1038/s41388-021-01811-8 33947958 PMC8357052

[B32] Grosso F. Jones R. L. Demetri G. D. Judson I. R. Blay J. Y. Le Cesne A. (2007). Efficacy of trabectedin (ecteinascidin-743) in advanced pretreated myxoid liposarcomas: a retrospective study. lancet Oncol. 8, 595–602. 10.1016/S1470-2045(07)70175-4 17586092

[B33] Grosso F. Sanfilippo R. Virdis E. Piovesan C. Collini P. Dileo P. (2009). Trabectedin in myxoid liposarcomas (MLS): a long-term analysis of a single-institution series. Ann. Oncol. 20, 1439–1444. 10.1093/annonc/mdp004 19465423

[B34] Haigis K. M. Sweet-Cordero A. (2011). New insights into oncogenic stress. Nat. Genet. 43, 177–178. 10.1038/ng0311-177 21350495

[B35] Harlow M. L. Maloney N. Roland J. Guillen Navarro M. J. Easton M. K. Kitchen-Goosen S. M. (2016). Lurbinectedin inactivates the ewing sarcoma oncoprotein EWS-FLI1 by redistributing it within the nucleus. Cancer Res. 76, 6657–6668. 10.1158/0008-5472.Can-16-0568 27697767 PMC5567825

[B36] Hartono A. B. Kang H. J. Shi L. Phipps W. Ungerleider N. Giardina A. (2022). Salt-inducible kinase 1 is a potential therapeutic target in desmoplastic small round cell tumor. Oncogenesis 11, 18. 10.1038/s41389-022-00395-6 35443736 PMC9021191

[B37] Hayes-Jordan A. LaQuaglia M. P. Modak S. (2016). Management of desmoplastic small round cell tumor. Semin. Pediatr. Surg. 25, 299–304. 10.1053/j.sempedsurg.2016.09.005 27955733 PMC5614508

[B38] Hingorani P. Dinu V. Zhang X. Lei H. Shern J. F. Park J. (2020). Transcriptome analysis of desmoplastic small round cell tumors identifies actionable therapeutic targets: a report from the Children's Oncology Group. Sci. Rep. 10, 12318. 10.1038/s41598-020-69015-w 32703985 PMC7378211

[B39] Hingorani P. Zhang W. Zhang Z. Xu Z. Wang W. L. Roth M. E. (2022). Trastuzumab deruxtecan, antibody-drug conjugate targeting HER2, is effective in pediatric malignancies: a report by the pediatric preclinical testing consortium. Mol. Cancer Ther. 21, 1318–1325. 10.1158/1535-7163.Mct-21-0758 35657346

[B40] Hollstein P. E. Eichner L. J. Brun S. N. Kamireddy A. Svensson R. U. Vera L. I. (2019). The AMPK-related kinases SIK1 and SIK3 mediate key tumor-suppressive effects of LKB1 in NSCLC. Cancer Discov. 9, 1606–1627. 10.1158/2159-8290.CD-18-1261 31350328 PMC6825547

[B41] Honoré C. Delhorme J. B. Nassif E. Faron M. Ferron G. Bompas E. (2019). Can we cure patients with abdominal Desmoplastic Small Round Cell Tumor? Results of a retrospective multicentric study on 100 patients. Surg. Oncol. 29, 107–112. 10.1016/j.suronc.2019.04.002 31196472

[B42] Hooijkaas A. Gadiot J. Morrow M. Stewart R. Schumacher T. Blank C. U. (2012). Selective BRAF inhibition decreases tumor-resident lymphocyte frequencies in a mouse model of human melanoma. Oncoimmunology 1, 609–617. 10.4161/onci.20226 22934253 PMC3429565

[B43] Hovsepyan S. Giani C. Pasquali S. Di Giannatale A. Chiaravalli S. Colombo C. (2023). Desmoplastic small round cell tumor: from state of the art to future clinical prospects. Expert Rev. Anticancer Ther. 23, 471–484. 10.1080/14737140.2023.2200171 37017324

[B44] Hsu J. Y. Seligson N. D. Hays J. L. Miles W. O. Chen J. L. (2022). Clinical utility of CDK4/6 inhibitors in sarcoma: successes and future challenges. JCO Precis. Oncol. 6, e2100211. 10.1200/po.21.00211 35108033 PMC8820917

[B45] Huang S. van Duijnhoven S. M. J. Sijts A. J. A. M. van Elsas A. (2020). Bispecific antibodies targeting dual tumor-associated antigens in cancer therapy. J. Cancer Res. Clin. Oncol. 146, 3111–3122. 10.1007/s00432-020-03404-6 32989604 PMC7679314

[B46] Kang H. J. Park J. H. Chen W. Kang S. I. Moroz K. Ladanyi M. (2014). EWS-WT1 oncoprotein activates neuronal reprogramming factor ASCL1 and promotes neural differentiation. Cancer Res. 74, 4526–4535. 10.1158/0008-5472.Can-13-3663 24934812

[B47] Kauffmann-Guerrero D. Huber R. M. (2020) “Orphan drugs in development for the treatment of small-cell lung cancer: emerging data on lurbinectedin,” in Lung cancer: targets and therapy, 27–31. 10.2147/LCTT.S239223 PMC7060031 32184690

[B48] Kennedy A. L. Vallurupalli M. Chen L. Crompton B. Cowley G. Vazquez F. (2015). Functional, chemical genomic, and super-enhancer screening identify sensitivity to cyclin D1/CDK4 pathway inhibition in Ewing sarcoma. Oncotarget 6, 30178–30193. 10.18632/oncotarget.4903 26337082 PMC4745789

[B49] Kim J. Lee K. Pelletier J. (1998). The desmoplastic small round cell tumor t(11;22) translocation produces EWS/WT1 isoforms with differing oncogenic properties. Oncogene 16, 1973–1979. 10.1038/sj.onc.1201716 9591781

[B50] Kontos F. Michelakos T. Kurokawa T. Sadagopan A. Schwab J. H. Ferrone C. R. (2021). B7-H3: an attractive target for antibody-based immunotherapy. Clin. Cancer Res. 27, 1227–1235. 10.1158/1078-0432.Ccr-20-2584 33051306 PMC7925343

[B51] Krejsgaard T. Vetter-Kauczok C. S. Woetmann A. Kneitz H. Eriksen K. W. Lovato P. (2009). Ectopic expression of B-lymphoid kinase in cutaneous T-cell lymphoma. Blood 113, 5896–5904. 10.1182/blood-2008-09-181024 19351960 PMC2700325

[B52] Kushner B. H. LaQuaglia M. P. Wollner N. Meyers P. A. Lindsley K. L. Ghavimi F. (1996). Desmoplastic small round-cell tumor: prolonged progression-free survival with aggressive multimodality therapy. J. Clin. Oncol. 14, 1526–1531. 10.1200/JCO.1996.14.5.1526 8622067

[B53] Ladanyi M. Gerald W. (1994). Fusion of the EWS and WT1 genes in the desmoplastic small round cell tumor. Cancer Res. 54, 2837–2840. 8187063

[B54] Lal D. R. Su W. T. Wolden S. L. Loh K. C. Modak S. La Quaglia M. P. (2005). Results of multimodal treatment for desmoplastic small round cell tumors. J. Pediatr. Surg. 40, 251–255. 10.1016/j.jpedsurg.2004.09.046 15868593

[B55] Lamhamedi-Cherradi S. E. Maitituoheti M. Menegaz B. A. Krishnan S. Vetter A. M. Camacho P. (2022). The androgen receptor is a therapeutic target in desmoplastic small round cell sarcoma. Nat. Commun. 13, 3057. 10.1038/s41467-022-30710-z 35650195 PMC9160255

[B56] Larson S. M. Carrasquillo J. A. Cheung N.-K. V. Press O. W. (2015). Radioimmunotherapy of human tumours. Nat. Rev. Cancer 15, 347–360. 10.1038/nrc3925 25998714 PMC4798425

[B57] Lelliott E. J. Sheppard K. E. McArthur G. A. (2022). Harnessing the immunotherapeutic potential of CDK4/6 inhibitors in melanoma: is timing everything? npj Precis. Oncol. 6, 26. 10.1038/s41698-022-00273-9 35444175 PMC9021218

[B58] Lettieri C. K. Garcia-Filion P. Hingorani P. (2014). Incidence and outcomes of desmoplastic small round cell tumor: results from the surveillance, epidemiology, and end results database. J. Cancer Epidemiol. 2014, 680126. 10.1155/2014/680126 25431592 PMC4238280

[B59] Liu C. Peng W. Xu C. Lou Y. Zhang M. Wargo J. A. (2013). BRAF inhibition increases tumor infiltration by T cells and enhances the antitumor activity of adoptive immunotherapy in mice. Clin. Cancer Res. 19, 393–403. 10.1158/1078-0432.Ccr-12-1626 23204132 PMC4120472

[B60] Long A. W. Xu H. Santich B. H. Guo H. Hoseini S. S. de Stanchina E. (2024). Heterodimerization of T cell engaging bispecific antibodies to enhance specificity against pancreatic ductal adenocarcinoma. J. Hematol. Oncol. 17, 20. 10.1186/s13045-024-01538-5 38650005 PMC11036555

[B61] Lowery C. D. Dowless M. Renschler M. Blosser W. VanWye A. B. Stephens J. R. (2019). Broad spectrum activity of the checkpoint kinase 1 inhibitor prexasertib as a single agent or chemopotentiator across a range of preclinical pediatric tumor models. Clin. Cancer Res. 25, 2278–2289. 10.1158/1078-0432.Ccr-18-2728 30563935 PMC6445779

[B62] Magrath J. W. Flinchum D. A. Hartono A. B. Goldberg I. N. Espinosa-Cotton M. Moroz K. (2023a). Genomic breakpoint characterization and transcriptome analysis of metastatic, recurrent desmoplastic small round cell tumor. Sarcoma 2023, 6686702. 10.1155/2023/6686702 37457440 PMC10344636

[B63] Magrath J. W. Flinchum D. A. Hartono A. B. Sampath S. S. O'Grady T. M. Baddoo M. (2024b). Transcriptomic analysis identifies B-lymphocyte kinase as a therapeutic target for desmoplastic small round cell tumor cancer stem cell-like cells. Oncogenesis 13, 2. 10.1038/s41389-023-00504-z 38177125 PMC10767073

[B64] Magrath J. W. Goldberg I. N. Truong D. D. Hartono A. B. Sampath S. S. Jackson C. E. (2023b). Enzalutamide induces cytotoxicity in desmoplastic small round cell tumor independent of the androgen receptor. bioRxiv 565842. 10.1101/2023.11.06.565842 PMC10995187 38575753

[B65] Magrath J. W. Goldberg I. N. Truong D. D. Hartono A. B. Sampath S. S. Jackson C. E. (2024c). Enzalutamide induces cytotoxicity in desmoplastic small round cell tumor independent of the androgen receptor. Commun. Biol. 7, 411. 10.1038/s42003-024-06003-0 38575753 PMC10995187

[B66] Magrath J. W. Kang H. J. Hartono A. Espinosa-Cotton M. Somwar R. Ladanyi M. (2022). Desmoplastic small round cell tumor cancer stem cell-like cells resist chemotherapy but remain dependent on the EWSR1-WT1 oncoprotein. Front. Cell. Dev. Biol. 10, 1048709. 10.3389/fcell.2022.1048709 36506091 PMC9732033

[B67] Magrath J. W. Sampath S. S. Flinchum D. A. Hartono A. B. Goldberg I. N. Boehling J. R. (2024a). Comprehensive transcriptomic analysis of EWSR1::WT1 targets identifies CDK4/6 inhibitors as an effective therapy for desmoplastic small round cell tumors. Cancer Res. 84, 1426–1442. 10.1158/0008-5472.Can-23-3334 38588409 PMC11063761

[B68] Malek S. N. Dordai D. I. Reim J. Dintzis H. Desiderio S. (1998). Malignant transformation of early lymphoid progenitors in mice expressing an activated Blk tyrosine kinase. Proc. Natl. Acad. Sci. U. S. A. 95, 7351–7356. 10.1073/pnas.95.13.7351 9636152 PMC22613

[B69] Modak S. Gerald W. Cheung N. K. (2002). Disialoganglioside GD2 and a novel tumor antigen: potential targets for immunotherapy of desmoplastic small round cell tumor. Med. Pediatr. Oncol. 39, 547–551. 10.1002/mpo.10151 12376975

[B70] Modak S. Zanzonico P. Grkovski M. Slotkin E. K. Carrasquillo J. A. Lyashchenko S. K. (2020). B7H3-Directed intraperitoneal radioimmunotherapy with radioiodinated omburtamab for desmoplastic small round cell tumor and other peritoneal tumors: results of a phase I study. J. Clin. Oncol. 38, JCO2001974–4291. 10.1200/jco.20.01974 PMC7768336 33119478

[B71] Monteran L. Erez N. (2019). The dark side of fibroblasts: cancer-associated fibroblasts as mediators of immunosuppression in the tumor microenvironment. Front. Immunol. 10, 1835. 10.3389/fimmu.2019.01835 31428105 PMC6688105

[B72] Myo Min K. K. Ffrench C. B. Jessup C. F. Shepherdson M. Barreto S. G. Bonder C. S. (2023). Overcoming the fibrotic fortress in pancreatic ductal adenocarcinoma: challenges and opportunities. Cancers (Basel) 15, 2354. 10.3390/cancers15082354 37190281 PMC10137060

[B73] Nakano K. (2023). The future of HER2-targeted treatment for osteosarcoma: lessons from the negative trastuzumab deruxtecan results. Int. J. Mol. Sci. 24, 16823. 10.3390/ijms242316823 38069146 PMC10706029

[B74] Negri T. Brich S. Bozzi F. Volpi C. V. Gualeni A. V. Stacchiotti S. (2017). New transcriptional-based insights into the pathogenesis of desmoplastic small round cell tumors (DSRCTs). Oncotarget 8, 32492–32504. 10.18632/oncotarget.16477 28415643 PMC5464804

[B75] Nicholas T. R. Metcalf S. A. Greulich B. M. Hollenhorst P. C. (2021). Androgen signaling connects short isoform production to breakpoint formation at Ewing sarcoma breakpoint region 1. Nar. Cancer 3, zcab033. 10.1093/narcan/zcab033 34409300 PMC8364332

[B76] Nishio J. Iwasaki H. Ishiguro M. Ohjimi Y. Fujita C. Yanai F. (2002). Establishment and characterization of a novel human desmoplastic small round cell tumor cell line, JN-DSRCT-1. Lab. Investig. 82, 1175–1182. 10.1097/01.lab.0000028059.92642.03 12218078

[B77] Ogura K. Somwar R. Hmeljak J. Magnan H. Benayed R. Momeni-Boroujeni A. (2021). Therapeutic potential of NTRK3 inhibition in desmoplastic small round cell tumor. Clin. Cancer Res. 27, 1184–1194. 10.1158/1078-0432.Ccr-20-2585 33229458 PMC8212565

[B78] Park J. A. Cheung N. V. (2022). Overcoming tumor heterogeneity by *ex vivo* arming of T cells using multiple bispecific antibodies. J. Immunother. Cancer 10, e003771. 10.1136/jitc-2021-003771 35086947 PMC8796264

[B79] Park J. A. Santich B. H. Xu H. Lum L. G. Cheung N. V. (2021). Potent *ex vivo* armed T cells using recombinant bispecific antibodies for adoptive immunotherapy with reduced cytokine release. J. Immunother. Cancer 9, e002222. 10.1136/jitc-2020-002222 33986124 PMC8126293

[B80] Patra K. C. Kato Y. Mizukami Y. Widholz S. Boukhali M. Revenco I. (2018). Mutant GNAS drives pancreatic tumourigenesis by inducing PKA-mediated SIK suppression and reprogramming lipid metabolism. Nat. Cell. Biol. 20, 811–822. 10.1038/s41556-018-0122-3 29941929 PMC6044476

[B81] Petersen D. L. Berthelsen J. Willerslev-Olsen A. Fredholm S. Dabelsteen S. Bonefeld C. M. (2017). A novel BLK-induced tumor model. Tumour Biol. 39, 1010428317714196. 10.1177/1010428317714196 28670978

[B82] Petersen D. L. Krejsgaard T. Berthelsen J. Fredholm S. Willerslev-Olsen A. Sibbesen N. A. (2014). B-lymphoid tyrosine kinase (Blk) is an oncogene and a potential target for therapy with dasatinib in cutaneous T-cell lymphoma (CTCL). Leukemia 28, 2109–2112. 10.1038/leu.2014.192 24919804 PMC4190403

[B83] Potluri S. Assi S. A. Chin P. S. Coleman D. J. L. Pickin A. Moriya S. (2021). Isoform-specific and signaling-dependent propagation of acute myeloid leukemia by Wilms tumor 1. Cell. Rep. 35, 109010. 10.1016/j.celrep.2021.109010 33882316

[B84] Rader J. Russell M. R. Hart L. S. Nakazawa M. S. Belcastro L. T. Martinez D. (2013). Dual CDK4/CDK6 inhibition induces cell-cycle arrest and senescence in neuroblastoma. Clin. Cancer Res. 19, 6173–6182. 10.1158/1078-0432.Ccr-13-1675 24045179 PMC3844928

[B85] Sakamoto K. Bultot L. Göransson O. (2018). The salt-inducible kinases: emerging metabolic regulators. Trends Endocrinol. Metabolism 29, 827–840. 10.1016/j.tem.2018.09.007 30385008

[B86] Sanchez Y. Wong C. Thoma R. S. Richman R. Wu Z. Piwnica-Worms H. (1997). Conservation of the Chk1 checkpoint pathway in mammals: linkage of DNA damage to Cdk regulation through Cdc25. Science 277, 1497–1501. 10.1126/science.277.5331.1497 9278511

[B87] Santich B. H. Cheal S. M. Ahmed M. McDevitt M. R. Ouerfelli O. Yang G. (2021). A self-assembling and disassembling (SADA) bispecific antibody (BsAb) platform for curative two-step pretargeted radioimmunotherapy. Clin. Cancer Res. 27, 532–541. 10.1158/1078-0432.Ccr-20-2150 32958698 PMC7855367

[B88] Saouaf S. J. Mahajan S. Rowley R. B. Kut S. A. Fargnoli J. Burkhardt A. L. (1994). Temporal differences in the activation of three classes of non-transmembrane protein tyrosine kinases following B-cell antigen receptor surface engagement. Proc. Natl. Acad. Sci. U. S. A. 91, 9524–9528. 10.1073/pnas.91.20.9524 7524079 PMC44845

[B89] Sato Y. Nabeta Y. Tsukahara T. Hirohashi Y. Syunsui R. Maeda A. (2002). Detection and induction of CTLs specific for SYT-SSX-derived peptides in HLA-A24(+) patients with synovial sarcoma. J. Immunol. 169, 1611–1618. 10.4049/jimmunol.169.3.1611 12133991

[B90] Scaltriti M. Verma C. Guzman M. Jimenez J. Parra J. L. Pedersen K. (2009). Lapatinib, a HER2 tyrosine kinase inhibitor, induces stabilization and accumulation of HER2 and potentiates trastuzumab-dependent cell cytotoxicity. Oncogene 28, 803–814. 10.1038/onc.2008.432 19060928

[B91] Schöffski P. Bahleda R. Wagner A. J. Burgess M. A. Junker N. Chisamore M. (2023). Results of an open-label, phase ia/b study of pembrolizumab plus olaratumab in patients with unresectable, locally advanced, or metastatic soft-tissue sarcoma. Clin. Cancer Res. 29, 3320–3328. 10.1158/1078-0432.Ccr-23-0742 37382656 PMC10472093

[B92] Shiravand Y. Khodadadi F. Kashani S. M. A. Hosseini-Fard S. R. Hosseini S. Sadeghirad H. (2022). Immune checkpoint inhibitors in cancer therapy. Curr. Oncol. 29, 3044–3060. 10.3390/curroncol29050247 35621637 PMC9139602

[B93] Sigismund S. Avanzato D. Lanzetti L. (2018). Emerging functions of the EGFR in cancer. Mol. Oncol. 12, 3–20. 10.1002/1878-0261.12155 29124875 PMC5748484

[B94] Slotkin E. K. Bowman A. S. Levine M. F. Dela Cruz F. Coutinho D. F. Sanchez G. I. (2021a). Comprehensive molecular profiling of desmoplastic small round cell tumor. Mol. Cancer Res. 19, 1146–1155. 10.1158/1541-7786.Mcr-20-0722 33753552 PMC8293793

[B95] Slotkin E. K. Bowman A. S. Levine M. F. Dela Cruz F. Coutinho D. F. Sanchez G. I. (2021b). Comprehensive molecular profiling of desmoplastic small round cell tumor. Mol. Cancer Res. 19, 1146–1155. 10.1158/1541-7786.Mcr-20-0722 33753552 PMC8293793

[B96] Slotkin E. K. Mauguen A. Ortiz M. V. Dela Cruz F. S. O'Donohue T. Kinnaman M. D. (2022). A phase I/II study of prexasertib in combination with irinotecan in patients with relapsed/refractory desmoplastic small round cell tumor and rhabdomyosarcoma. J. Clin. Oncol. 40, 11503. 10.1200/JCO.2022.40.16_suppl.11503

[B97] Smith R. S. Odintsov I. Liu Z. Lui A. J. W. Hayashi T. Vojnic M. (2022). Novel patient-derived models of desmoplastic small round cell tumor confirm a targetable dependency on ERBB signaling. Dis. Model. Mech. 15, dmm047621. 10.1242/dmm.047621 34841430 PMC8807576

[B98] Sterner R. C. Sterner R. M. (2021). CAR-T cell therapy: current limitations and potential strategies. Blood Cancer J. 11, 69. 10.1038/s41408-021-00459-7 33824268 PMC8024391

[B99] Stiles Z. E. Dickson P. V. Glazer E. S. Murphy A. J. Davidoff A. M. Behrman S. W. (2018). Desmoplastic small round cell tumor: a nationwide study of a rare sarcoma. J. Surg. Oncol. 117, 1759–1767. 10.1002/jso.25071 29878371

[B100] Subbiah V. Lamhamedi-Cherradi S. E. Cuglievan B. Menegaz B. A. Camacho P. Huh W. (2018). Multimodality treatment of desmoplastic small round cell tumor: chemotherapy and complete cytoreductive surgery improve patient survival. Clin. Cancer Res. 24, 4865–4873. 10.1158/1078-0432.Ccr-18-0202 29871905 PMC6168359

[B101] Tabak S. A. Khalifa S. E. Fathy Y. (2018). HER-2 immunohistochemical expression in bone sarcomas: a new hope for osteosarcoma patients. Open Access Maced. J. Med. Sci. 6, 1555–1560. 10.3889/oamjms.2018.318 30337965 PMC6182517

[B102] Talukdar S. Emdad L. Das S. K. Fisher P. B. (2020). EGFR: an essential receptor tyrosine kinase-regulator of cancer stem cells. Adv. Cancer Res. 147, 161–188. 10.1016/bs.acr.2020.04.003 32593400

[B103] Tarumoto Y. Lu B. Somerville T. D. D. Huang Y. H. Milazzo J. P. Wu X. S. (2018). LKB1, salt-inducible kinases, and MEF2C are linked dependencies in acute myeloid leukemia. Mol. Cell. 69, 1017–1027. 10.1016/j.molcel.2018.02.011 29526696 PMC5856641

[B104] Tawbi H. A. Burgess M. Bolejack V. Van Tine B. A. Schuetze S. M. Hu J. (2017). Pembrolizumab in advanced soft-tissue sarcoma and bone sarcoma (SARC028): a multicentre, two-cohort, single-arm, open-label, phase 2 trial. Lancet Oncol. 18, 1493–1501. 10.1016/s1470-2045(17)30624-1 28988646 PMC7939029

[B105] Teplinsky E. Herzog T. J. (2017). The efficacy of trabectedin in treating ovarian cancer. Expert Opin. Pharmacother. 18, 313–323. 10.1080/14656566.2017.1285282 28140689

[B106] Terry R. L. Meyran D. Fleuren E. D. G. Mayoh C. Zhu J. Omer N. (2021). Chimeric antigen receptor T cell therapy and the immunosuppressive tumor microenvironment in pediatric sarcoma. Cancers (Basel) 13, 4704. 10.3390/cancers13184704 34572932 PMC8465026

[B107] Texido G. Su I. H. Mecklenbräuker I. Saijo K. Malek S. N. Desiderio S. (2000). The B-cell-specific Src-family kinase Blk is dispensable for B-cell development and activation. Mol. Cell. Biol. 20, 1227–1233. 10.1128/mcb.20.4.1227-1233.2000 10648608 PMC85250

[B108] Truong D. Magrath J. Lee S. Ludwig J. (2024). Abstract 2851: characterization of neogenes in desmoplastic small round cell tumors. Cancer Res. 84, 2851. 10.1158/1538-7445.Am2024-2851

[B109] Truong D. D. Lamhamedi-Cherradi S. E. Porter R. W. Krishnan S. Swaminathan J. Gibson A. (2023). Dissociation protocols used for sarcoma tissues bias the transcriptome observed in single-cell and single-nucleus RNA sequencing. BMC Cancer 23, 488. 10.1186/s12885-023-10977-1 37254069 PMC10230784

[B110] Turner N. C. Slamon D. J. Ro J. Bondarenko I. Im S. A. Masuda N. (2018). Overall survival with palbociclib and fulvestrant in advanced breast cancer. N. Engl. J. Med. 379, 1926–1936. 10.1056/NEJMoa1810527 30345905

[B111] Uboldi S. Craparotta I. Colella G. Ronchetti E. Beltrame L. Vicario S. (2017). Mechanism of action of trabectedin in desmoplastic small round cell tumor cells. BMC Cancer 17, 107. 10.1186/s12885-017-3091-1 28166781 PMC5294815

[B112] Uribe M. L. Marrocco I. Yarden Y. (2021). EGFR in cancer: signaling mechanisms, drugs, and acquired resistance. Cancers (Basel) 13, 2748. 10.3390/cancers13112748 34206026 PMC8197917

[B113] VanArsdale T. Boshoff C. Arndt K. T. Abraham R. T. (2015). Molecular pathways: targeting the cyclin D-CDK4/6 Axis for cancer treatment. Clin. Cancer Res. 21, 2905–2910. 10.1158/1078-0432.Ccr-14-0816 25941111

[B114] van Erp A. E. M. Hillebrandt-Roeffen M. H. S. van Bree N. F. H. N. Plüm T. A. Flucke U. E. Desar I. M. E. (2022). Targeting the FAK-src complex in desmoplastic small round cell tumors, ewing sarcoma, and rhabdomyosarcoma. Sarcoma 2022, 3089424. 10.1155/2022/3089424 35655525 PMC9153931

[B115] van Erp A. E. M. van Houdt L. Hillebrandt-Roeffen M. H. S. van Bree N. F. H. N. Flucke U. E. Mentzel T. (2020). Olaparib and temozolomide in desmoplastic small round cell tumors: a promising combination *in vitro* and *in vivo* . J. Cancer Res. Clin. Oncol. 146, 1659–1670. 10.1007/s00432-020-03211-z 32279088 PMC7256072

[B116] Vibert J. Saulnier O. Collin C. Petit F. Borgman K. J. E. Vigneau J. (2022). Oncogenic chimeric transcription factors drive tumor-specific transcription, processing, and translation of silent genomic regions. Mol. Cell. 82, 2458–2471.e9. 10.1016/j.molcel.2022.04.019 35550257

[B117] Wang L. Liang C. Li F. Guan D. Wu X. Fu X. (2017). PARP1 in carcinomas and PARP1 inhibitors as antineoplastic drugs. Int. J. Mol. Sci. 18, 2111. 10.3390/ijms18102111 28991194 PMC5666793

[B118] Wang Y. Shi T. Song X. Liu B. Wei J. (2021). Gene fusion neoantigens: emerging targets for cancer immunotherapy. Cancer Lett. 506, 45–54. 10.1016/j.canlet.2021.02.023 33675984

[B119] Waqar S. H. B. Ali H. (2022). Changing incidence and survival of desmoplastic small round cell tumor in the USA. Proc. Bayl Univ. Med. Cent. 35, 415–419. 10.1080/08998280.2022.2049581 35754588 PMC9196647

[B120] Wilmott J. S. Long G. V. Howle J. R. Haydu L. E. Sharma R. N. Thompson J. F. (2012). Selective BRAF inhibitors induce marked T-cell infiltration into human metastatic melanoma. Clin. Cancer Res. 18, 1386–1394. 10.1158/1078-0432.Ccr-11-2479 22156613

[B121] Wu C. C. Beird H. C. Lamhamedi-Cherradi S. E. Soeung M. Ingram D. Truong D. D. (2022a). Multi-site desmoplastic small round cell tumors are genetically related and immune-cold. NPJ Precis. Oncol. 6, 21. 10.1038/s41698-022-00257-9 35379887 PMC8980094

[B122] Wu C.-C. Beird H. C. Lamhamedi-Cherradi S. E. Soeung M. Ingram D. Truong D. D. (2022b). Multi-site desmoplastic small round cell tumors are genetically related and immune-cold. npj Precis. Oncol. 6, 21. 10.1038/s41698-022-00257-9 35379887 PMC8980094

[B123] Xiang T. Zhang S. Y. Wang S. S. Fei R. S. Li H. (2020). A nationwide analysis of desmoplastic small round cell tumor. Med. Baltim. 99, e21337. 10.1097/md.0000000000021337 PMC7387032 32791732

[B124] Yang W. Lee K. W. Srivastava R. M. Kuo F. Krishna C. Chowell D. (2019). Immunogenic neoantigens derived from gene fusions stimulate T cell responses. Nat. Med. 25, 767–775. 10.1038/s41591-019-0434-2 31011208 PMC6558662

[B125] Yotnda P. Firat H. Garcia-Pons F. Garcia Z. Gourru G. Vernant J. P. (1998). Cytotoxic T cell response against the chimeric p210 BCR-ABL protein in patients with chronic myelogenous leukemia. J. Clin. Investig. 101, 2290–2296. 10.1172/jci488 9593785 PMC508817

[B126] Zhang P. J. Goldblum J. R. Pawel B. R. Fisher C. Pasha T. L. Barr F. G. (2003). Immunophenotype of desmoplastic small round cell tumors as detected in cases with EWS-WT1 gene fusion product. Mod. Pathol. 16, 229–235. 10.1097/01.Mp.0000056630.76035.F3 12640103

[B127] Zhu L. Keck J. Stommel J. Johnson B. Corless C. L. Ryan C. W. (2023). Deep multi-omic analyses to identify targetable pathways in desmoplastic small round cell tumor (DSRCT) with opportunities for clinical intervention. J. Clin. Oncol. 41, e22006. 10.1200/JCO.2023.41.16_suppl.e22006

[B128] Zuco V. Pasquali S. Tortoreto M. Percio S. Doldi V. Barisella M. (2023a). Effectiveness of irinotecan plus trabectedin on a desmoplastic small round cell tumor patient-derived xenograft. Dis. Model. Mech. 16, dmm049649. 10.1242/dmm.049649 37158111 PMC10281256

[B129] Zuco V. Pasquali S. Tortoreto M. Percio S. Doldi V. Barisella M. (2023b). Effectiveness of irinotecan plus trabectedin on a desmoplastic small round cell tumor patient-derived xenograft. Dis. Models Mech. 16, dmm049649. 10.1242/dmm.049649 PMC10281256 37158111

